# Marked attenuation of inflammatory mediator-induced C-fiber sensitization for mechanical and hypotonic stimuli in TRPV4^-/- ^mice

**DOI:** 10.1186/1744-8069-3-31

**Published:** 2007-10-29

**Authors:** Xiaojie Chen, Nicole Alessandri-Haber, Jon D Levine

**Affiliations:** 1 Departments of Anatomy, Medicine and Oral and Maxillofacial Surgery, Division of Neuroscience, University of California, San Francisco, CA 94143, USA

## Abstract

Inflammatory mediators can directly sensitize primary afferent nociceptors to mechanical and osmotic stimuli. Sensitized nociceptors have a lowered threshold of activation and increased spontaneous activity, which result in symptoms of hyperalgesia and pain, respectively. The transient receptor potential vanilloid 4 (TRPV4) ligand-gated ion channel has been implicated in the hyperalgesia for mechanical and osmotic stimuli associated with inflammatory states. To investigate whether TRPV4 directly contributes to the mechanisms of inflammatory mediator sensitization of C-fiber nociceptors, we compared the effect of the injection of simplified inflammatory soup (prostaglandin E_2 _and serotonin) into the mechanical receptive fields of C-fibers in TRPV4^+/+ ^and TRPV4^-/- ^mice *in vivo*. Following the injection of the soup, the percentage of C-fibers responding to a hypotonic stimulus and the magnitude of the response was significantly greater in TRPV4^+/+ ^mice compared to TRPV4^-/- ^mice. Moreover, in response to simplified inflammatory soup only C-fibers from TRPV4^+/+ ^mice exhibited increased spontaneous activity and decreased mechanical threshold. These marked impairments in the response of C-fibers in TRPV4^-/- ^mice demonstrate the importance of TRPV4 in nociceptor sensitization; we suggest that TRPV4, as TRPV1, underlies the nociceptive effects of multiple inflammatory mediators on primary afferent.

## Background

Transient receptor potential vanilloid 4 (TRPV4), a member of the vanilloid subfamily of transient receptor potential ligand-gated ion channels, cloned from hypothalamus using a functional assay screening for osmo-sensitivity [1] or kidney [2], is also present in sensory neurons that express properties of nociceptors [3,4]. Accumulating data support a role of TRPV4 in nociception: 1) mice lacking a functional TRPV4 gene show impaired responses to intense noxious mechanical stimuli but normal responses to low-threshold mechanical stimuli [5,6], 2) TRPV4 plays an important role in hyperalgesia to osmotic and mechanical stimuli generated by inflammatory mediators [7,8], and 3) inflammatory mediators can engage TRPV4 in hyperalgesia to mechanical and osmotic stimuli [9]. While primary afferent nociceptors in the rat respond to hypotonic stimuli, an effect that is enhanced by prostaglandin E_2 _[7] on the role of TRPV4 is unknown. To establish the role of TRPV4, *in vivo*, in peripheral nociceptor sensitization, we performed a single fiber electrophysiology study of primary afferent nociceptors in TRPV4^+/+ ^and TRPV4^-/- ^mice.

## Results

There were no significant differences in the average conduction velocity and baseline mechanical threshold for C-fibers from TRPV4^+/+ ^and TRPV4^-/- ^mice (unpaired *t*- and Mann Whitney test, respectively, both p > 0.05). The average conduction velocity of C-fibers from TRPV4^+/+ ^and TRPV4^-/- ^mice were 1.1 ± 0.1 and 1.0 ± 0.1 m/sec, respectively. And the average baseline mechanical threshold of C-fibers from TRPV4^+/+ ^and TRPV4^-/- ^mice were 23.7 ± 7.86 and 16.2 ± 5.73 mN, respectively. Their receptive fields were both approximately 2 mm across. However, in TRPV4^-/- ^mice C-fiber spontaneous activity was 4.15 ± 1.61 spikes/min, which was significantly higher than in TRPV4^+/+ ^controls (0.18 ± 0.18 spikes/min, unpaired *t*-test, p < 0.05). Of note, only one C-fiber from a TRPV4^+/+ ^mouse had spontaneous activity, at a very low frequency (2 spikes/min), while 38.5% (5/13) of C-fibers from TRPV4^-/- ^mice had low frequency spontaneous activity (average, 11 spikes/min, n = 5, p < 0.05).

**Figure 1:**
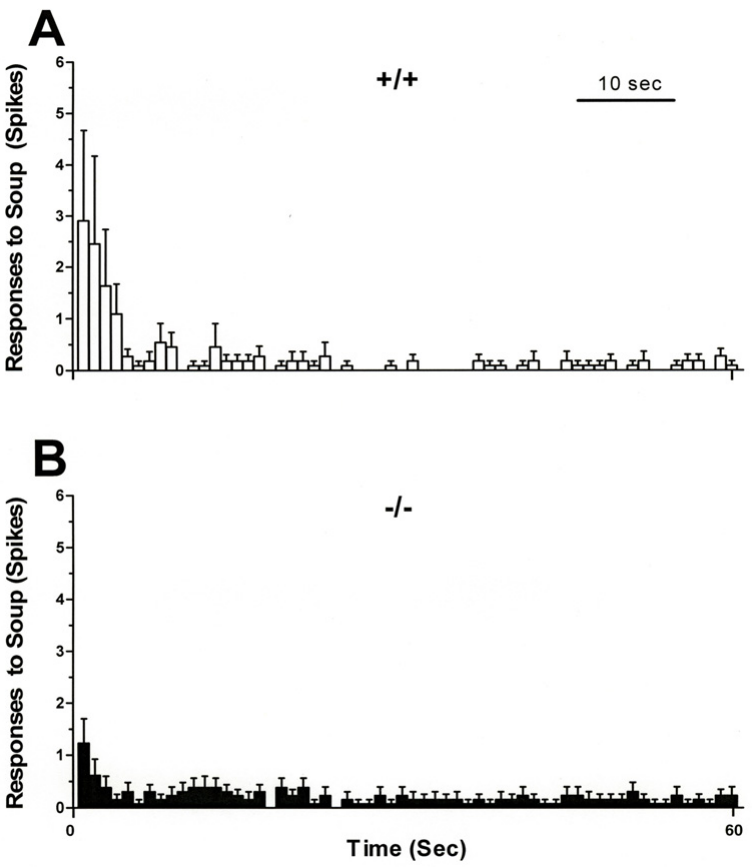
The average time course of the response of C-fibers during the 60 sec period after injection of simplified inflammatory soup was not significantly different in TRPV4^+/+ ^and TRPV4^-/- ^mice (unpaired *t*-test, p > 0.05). The bin width is 1 sec. **A**. The open bars represent activity evoked by simplified inflammatory soup in TRPV4^+/+ ^C-fibers (n = 11), and **B**. The filled bars represent action potentials evoked in TRPV4^-/- ^C-fibers (n = 13).

Approximately half of C-fibers in both TRPV4^+/+ ^and TRPV4^-/- ^mice were excited by intradermal injection of simplified inflammatory soup, adjacent to their mechanical receptive fields. The magnitude of activity evoked by injection of simplified inflammatory soup was similar between TRPV4^+/+ ^and TRPV4^-/- ^mice (p = ns, Fig. 1).

**Figure 2:**
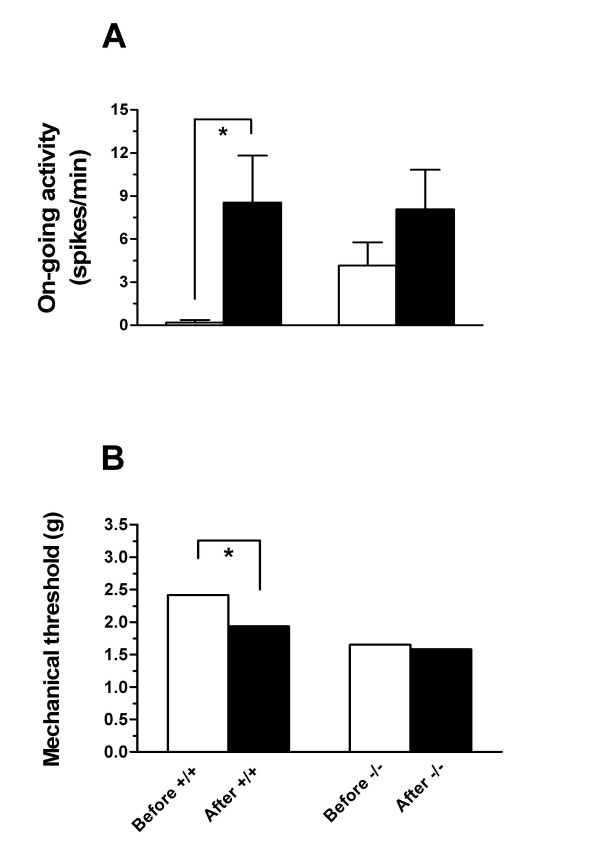
**A**. On-going activity in C-fibers before (open bar) and after (filled bar) injection of simplified inflammatory soup into each fiber's mechanical receptive field was significantly increased in TRPV4^+/+ ^(n = 11, paired *t*-test, * p < 0.05) but not TRPV4^-/- ^mice (n = 13, paired *t*-test, p > 0.05). **B**. The mechanical threshold of C-fibers in TRPV4^+/+ ^(open bar, n = 11) produced by intradermal injection of simplified inflammatory soup was statistically significant (Wilcoxon matched test, * p < 0.05). However, simplified inflammatory soup did not significantly change mechanical threshold of C-fibers in TRPV4^-/- ^mice (filled bars, n = 13, Wilcoxon matched test, p > 0.05). The change in mechanical threshold of C-fibers after simplified inflammatory soup was significantly greater in TRPV4^+/+ ^than TRPV4^-/- ^C-fibers (*χ*^2 ^test, * p < 0.05).

Almost all C-fibers from TRPV4^+/+ ^mice had no spontaneous activity before applying stimuli on the receptive fields, except one fiber with very low frequency of on-going activity (2 spikes/min). While baseline spontaneous activity was greater in TRPV4^-/- ^mice, on-going activity after injection of simplified inflammatory soup was significantly increased in TRPV4^+/+ ^C-fibers (p < 0.05), while it was unchanged in the TRPV4^-/- ^C-fibers (p = ns, Fig. 2A). Also, while there was no difference in baseline threshold between C-fibers in TRPV4^+/+ ^and TRPV4^-/- ^mice, after injection of simplified inflammatory soup, the mechanical threshold of C-fibers in TRPV4^+/+ ^but not TRPV4^-/- ^mice significantly decreased (p < 0.05, Fig. 2B).

**Figure 3:**
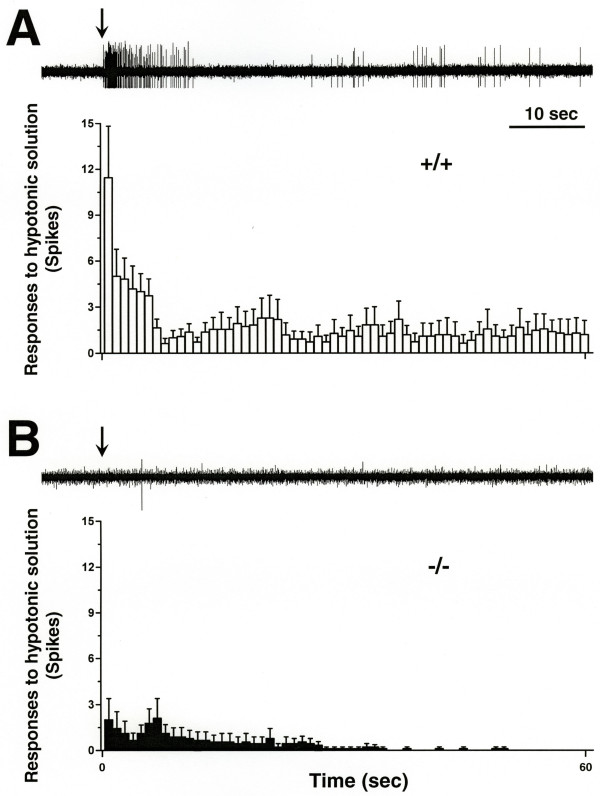
*Upper panel*, response of a TRPV4^+/+ ^C-fiber to hypotonic solution 15 min after injection of simplified inflammatory soup. Arrow indicates the time of injection of hypotonic solution. *Lower panel*, the average time course of the response of C-fibers during the first 60 sec after injection of hypotonic solution in TRPV4^+/+ ^mice (open bar, n = 11). The bin width is 1 sec. **B**. *Upper panel*, response of a TRPV4^-/- ^C-fiber to hypotonic solution after injection of simplified inflammatory soup. *Lower panel*, the average time course of the response of C-fibers during the first 60 sec after injection of hypotonic solution in TRPV4^-/- ^mice (filled bars, n = 9).

Finally, in TRPV4^+/+ ^mice, 81.2% (9/11) of C-fibers responded to intradermal hypotonic solution injected 15 minutes after intradermal injection of simplified inflammatory soup; only 33.3% (3/9) of C-fibers responded in TRPV4^-/- ^mice (Fig. 3, p < 0.05).

## Discussion

Recent studies support a role of TRPV4 in inflammatory mediator-induced sensitization to osmotic and mechanical stimuli: 1) TRPV4 contributes to the enhanced nociceptive behaviors in response to mechanical and osmotic stimuli, produced by inflammation and peripheral neuropathy [4,8], 2) inflammatory mediators can engage TRPV4 in DRG neurons *in vitro *[9] and 3) hypotonic stimuli elicit action potentials in C-fibers, and in presence of inflammatory mediators, the number of action potentials significantly increases, as does the percentage of responsive fibers [7]. To establish the contribution of TRPV4 in inflammatory mediator-induced sensitization of C-fiber nociceptors, *in vivo*, we compared the effect of inflammatory mediators that engage TRPV4 function in behavioral studies, on the response of C-fibers to osmotic and mechanical stimulation in TRPV4^+/+ ^and TRPV4^-/- ^mice. The percentage of C-fibers responding to hypotonicity as well as the magnitude of the response, is significantly greater in TRPV4^+/+ ^but not TRPV4^-/- ^mice (Fig. 3). This finding demonstrates a contribution of TRPV4 to inflammatory mediator-induced sensitization of C-fibers to hypotonic stimuli. This finding also has clinical relevance since water is an irritant, exacerbating painful conditions [10-12], and changes in tissue osmolarity have been reported in diseases that are associated with painful peripheral neuropathy such as diabetes [13] and alcoholism [14].

However, recent reports suggest that TRPV4 may also act as a mechano-transducer in primary afferent nociceptors; mice lacking a functional TRPV4 gene have impaired behavioral responses to intense noxious mechanical stimuli but normal response to low-threshold mechanical stimuli [5,6], and spinal intrathecal administration of oligodeoxynucleotides antisense to TRPV4 reverses mechanical hyperalgesia in a rat model of small-fiber painful peripheral neuropathy induced by the cancer chemotherapy agent Taxol^® ^[4]. In addition, while the baseline mechanical paw-withdrawal threshold is not significantly different between TRPV4^+/+ ^and TRPV4^-/- ^mice, after intraplantar injection of simplified inflammatory soup, mechanical hyperalgesia only occurred in TRPV4^+/+ ^mice [9]. Similarly, mechanical hyperalgesia induced by simplified inflammatory soup, in the rat, is prevented by spinal intrathecal treatment with TRPV4 antisense [9]. These findings suggested a role for TRPV4 in inflammatory mediator-induced sensitization of nociceptors to mechanical stimuli. Our present study actually demonstrated, *in vivo*, the role of TRPV4 in nociceptor sensitization, the mechanism underlying primary mechanical hyperalgesia. In agreement with behavioral studies demonstrating similar mechanical nociceptive thresholds in TRPV4^+/+ ^and TRPV4^-/- ^mice [5,6,9], the mechanical thresholds of C-fibers from TRPV4^+/+ ^and TRPV4^-/- ^mice were not significantly different. However, intradermal injection of simplified inflammatory soup lowered mechanical threshold in TRPV4^+/+ ^but not TRPV4^-/- ^C-fibers, supporting the idea of an *in vivo *contribution of TRPV4 to inflammatory mediator-induced sensitization of primary afferent nociceptors to mechanical stimuli.

Inflammatory mediators, such as PGE_2 _and 5-HT act directly on primary afferent nociceptors, to decrease their threshold of activation, increase spontaneous activity and increase response to supra-threshold stimuli (for review) [15]; whereas spontaneous activity of peripheral nociceptors may contribute to ongoing pain [16]. In this regard, it is noteworthy that simplified inflammatory soup enhanced spontaneous activity in C-fibers from TRPV4^+/+ ^but not TRPV4^-/- ^mice. This result is also compatible with the recent suggestion that depending on the cellular context some TRP channels may mediate increases in neuronal excitability through activation of intracellular signaling pathways rather than through the binding of a specific ligand [17]. We recently demonstrated that the simplified inflammatory soup constituting two inflammatory mediators (PGE_2 _and 5-HT) can act synergistically through cAMP to engage TRPV4 in mechanical hyperalgesia [9]. The role of TRPV4 not only in mechanical and osmotic hyperalgesia but also in the inflammatory mediator-induced increase in spontaneous activity in primary afferent nociceptors suggests that TRPV4 contributes to peripheral sensitization via the nociceptive effects of multiple inflammatory mediators. Moreover, to our knowledge this is the first report suggesting a role for TRPV4 in any form of spontaneous pain.

Not previously reported, TRPV4 may also play a role in the regulation of spontaneous activity of nociceptors, even in the absence of inflammation; spontaneous activity of C-fibers is about 20-fold *higher *in TRPV4^-/- ^mice than in TRPV4^+/+ ^mice. Thus, low level of on-going activity in primary afferents may not always result in spontaneous pain. The significant change of primary afferent activities can be more important as we observed in TRPV4^+/+ ^mice after simplified inflammatory soup. Of note, C-fiber conduction velocity is similar in TRPV4^+/+ ^and TRPV4^-/- ^mice. Since nerve conduction velocity is a measure of the excitability of voltage-sensitive channels in neurons, a variation in its value would reflect compensatory changes in voltage-gated ion channels in the axon [18-20], the difference in spontaneous activity is less likely to result from compensatory changes in most of the voltage-gated channels found in axons, but may reflect a contribution of TRPV4 in the modulation of membrane potential and the generation of action potentials in primary afferent nociceptors. The contribution of TRPV4 to spontaneous activity in primary afferent nociceptors, both in physiological and inflammatory states, support findings in other cell types where TRPV4 alters cell function by modulating complex calcium events [21-23]. Of course, we cannot rule out the possibility that subtle compensatory abnormalities in ion channel function in the TRPV4^-/- ^mouse are responsible for the change of the spontaneous activity. Anyway, the actual mechanism of spontaneous activity of primary afferents in TRPV4^-/- ^mice remains to be further elucidated.

## Conclusion

Using *in vivo *single fiber electrophysiology, we demonstrate a role of TRPV4 in inflammatory mediator-induced sensitization of C-fibers to hypotonic and mechanical stimuli. Moreover, TRPV4 may play a broader role in inflammatory pain, also contributing to the spontaneous pain induced by the action of inflammatory mediators. Therefore, we suggest that TRPV4 may play a similar role as TRPV1 which underlines mechanical and thermal sensitivities in the sensitization of primary nociceptive afferent, acting as a final pathway for the nociceptive effects of multiple inflammatory mediators and second messenger signaling to mediate primary afferent nociceptor sensitization to mechanical stimuli and, therefore, mechanical hyperalgesia.

## Methods

### Animal model

Experiments were performed on 24–34 g adult male C57BL6 mice lacking a functional TRPV4 gene (TRPV4^-/-^) and TRPV4 wild-type (TRPV4^+/+^) littermates [5]. A controlled heater (TR-200, FST, Foster City, CA) was used to maintain body temperature (≥ 36.5°C). Animal care and use conformed to National Institutes of Health (NIH) guidelines and was approved by the UCSF Committee on Animal Research.

### Electrophysiology

*In vivo *single fiber electrophysiology experiments were performed as described previously in rats [24,25] and mice [26]. Briefly, mice were anesthetized with sodium pentobarbital (initially 50 mg/kg, i.p., with additional doses given throughout the experiment to maintain areflexia). Recordings were made from C-fibers in the saphenous nerve, which innervates part of the dorsal surface of the hind paw. Bipolar stimulating electrodes were placed under the nerve, distal to the recording site. The nerve was cut proximal to the recording site and put on a platform for further dissection. Fine fascicles of axons were teased from the nerve and placed on a recording electrode. There usually are more than one fiber in the fasicle from which our recordings are made (often two or three). Single units were first detected by electrical stimulation of the nerve. Each fiber's conduction velocity was calculated by dividing the distance between the stimulating and recording electrodes by the latency of the electrically-evoked action potential. Fibers that conducted slower than 2 m/s were classified as C-fibers [27,28]. C-fiber receptive fields were located using a mechanical search stimulus, either a blunt probe with smooth tip or a 60 g von Frey hair (VFH). The electrically evoked action potential corresponding to the C-fiber whose mechanical receptive field had been identified was verified by the latency delay technique, in which electrically evoked spikes resulted in longer latency when the receptive field of the same fiber was stimulated mechanically [29]. Mechanical threshold was determined with calibrated VFH and defined as the lowest force that elicited ≥ 2 spikes within 1 s, in at least 50% of trials.

The spontaneous activity was expressed in spikes/min and measured one minute before and after injection of soup, respectively. The neural activity of C-fibers was captured and stored by using an IBM-compatible computer with MICRO 1401 interface (CED, Cambridge, UK) and further analyzed with *Spike2 *software (CED). Generally, the studied unit is the largest unit which can be easily captured by a window discriminator (Winston Electronics, San Francisco, CA) or by *Spike2 *software according to the amplitude and duration of action potentials. At the end of the experiment the mouse was euthanized by sodium pentobarbital followed by bilateral thoracotomy.

### Injections of simplified inflammatory soup and hypotonic solution

The simplified inflammatory soup used in these experiments was composed of the combination of prostaglandin E_2 _(PGE_2_) and serotonin (5-HT), both purchased from Sigma (St. Louis, MO), dissolved in 0.9% saline. A 2.5 μl volume of simplified inflammatory soup containing 100 ng each of PGE_2 _and 5-HT, was injected adjacent to a C-fiber's mechanical receptive field, which produced mechanical hyperalgesia in behavioral studies [9]. Hypotonic solution (deionized distilled water, 2.5 μl) was injected into the receptive field of an identified C-fiber, 15 min after the simplified inflammatory soup; after the simplified inflammatory soup was injected, mechanical threshold was re-measured, before recording the response to re-injection of hypotonic solution.

### Statistics

Group data are expressed as mean ± SEM. Statistical analyses were done using paired or unpaired *t*-test, Wilcoxon matched or Mann Whitney and *χ*^2 ^test, as appropriate. Differences were considered significant at a p-value of <0.05.

## Authors' contributions

XC participated in the design of the study, carried out all the experiment, performed the statistical analysis and drafted the manuscript. NA bred the mice and joined drafting the manuscript. JDL participated in the design of the study and drafted the manuscript. All authors read and approved the final manuscript.
